# *Spondias mombin* supplementation attenuated cardiac remodelling process induced by tobacco smoke

**DOI:** 10.1111/jcmm.13683

**Published:** 2018-05-29

**Authors:** Maria Angélica Martins Lourenço, Mariana Gobbo Braz, Aline Garcia Aun, Bruna Letícia Buzati Pereira, Amanda Menezes Figueiredo, Renata Aparecida Cândido da Silva, Elisa Moya Kazmarek, Patrícia Helena Correa Alegre, Tatiana Fernanda Bachiega, Silmeia Garcia Zanati, Paula Schmidt Azevedo, Bertha Furlan Polegato, Ana Angélica Henrique Fernandes, Sergio Alberto Rupp de Paiva, Leonardo Antonio Mamede Zornoff, Marcos Ferreira Minicucci

**Affiliations:** ^1^ Internal Medicine Department Botucatu Medical School UNESP—São Paulo State University Botucatu Brazil; ^2^ Department of Anesthesiology Botucatu Medical School UNESP—São Paulo State University Botucatu Brazil; ^3^ Chemistry and Biochemistry Department Institute of Biological Sciences Botucatu Medical School UNESP—São Paulo State University Botucatu Brazil

**Keywords:** cardiac remodelling, cigarette smoke, energy metabolism, fruit, oxidative stress

## Abstract

The objective of this study was to investigate the influence of *Spondias mombin* (SM) supplementation on the cardiac remodelling process induced by exposure to tobacco smoke (ETS) in rats. Male Wistar rats were divided into 4 groups: group C (control, n = 20) comprised animals not exposed to cigarette smoke and received standard chow; group ETS (n = 20) comprised animals exposed to cigarette smoke and received standard chow; group ETS100 (n = 20) received standard chow supplemented with 100 mg/kg body weight/d of SM; and group ETS250 (n = 20) received standard chow supplemented with 250 mg/kg body weight/d of SM. The observation period was 2 months. The ETS animals had higher values of left cardiac chamber diameters and of left ventricular mass index. SM supplementation attenuated these changes. In addition, the myocyte cross‐sectional area (CSA) was lower in group C compared with the ETS groups; however, the ETS250 group had lower values of CSA compared with the ETS group. The ETS group also showed higher cardiac levels of lipid hydroperoxide (LH) compared with group C; and, groups ETS100 and ETS250 had lower concentrations of LH compared with the ETS group. Regarding energy metabolism, SM supplementation decreased glycolysis and increased the β‐oxidation and the oxidative phosphorylation. There were no differences in the expression of Nrf‐2, SIRT‐1, NF‐κB, interferon‐gamma and interleukin 10. In conclusion, our results suggest that ETS induced the cardiac remodelling process. In addition, SM supplementation attenuated this process, along with oxidative stress reduction and energy metabolism modulation.

## 1 INTRODUCTION

Exposure to tobacco smoke is considered the most important cause of preventable death in the world.^1^ It is responsible for more than 6 million deaths per year and, according to some estimates, in 2020, it will cause more than 10 million deaths per year.^2^ In the cardiovascular system, ETS is a known risk factor for atherosclerosis, endothelial dysfunction, acute coronary syndromes and sudden death.^3^, ^4^ However, some clinical and experimental studies evaluated the direct effects of tobacco smoke on the heart, independently of atherosclerosis or hypertension.^5^, ^6^, ^7^, ^8^ These studies showed that ETS induces cardiac hypertrophy, increases left cardiac chambers and leads to ventricular dysfunction.^9^, ^10^, ^11^ These alterations are characteristics of the cardiac remodelling process. According to Cohn et al,^12^ the cardiac remodelling may be defined as genome expression, molecular, cellular and interstitial changes that are manifested clinically as changes in size, shape and function of the heart after cardiac injury. In the acute phase of cardiac injury, remodelling is an adaptive process enabling the heart to maintain function; however, chronically it leads to heart failure and death.^12^

Experimental studies showed that lipotoxicity, oxidative stress, inflammation, hemodynamic overload and increased expression of mitogen‐activated protein kinases (MAPK) are important mechanistic pathways linked with the effects of tobacco smoke on cardiac remodelling.^13^, ^14^, ^15^, ^16^ To attenuate this process, the supplementation of antioxidant and anti‐inflammatory substances, such as vitamin D, beta‐carotene, retinoic acid and taurine, has been studied.^17^, ^18^, ^19^, ^20^ In this scenario, SM deserves attention due to its antioxidant and anti‐inflammatory properties.

*Spondias mombin* is a tropical fruit that belongs to the family Anacardiaceae. The fruit is a drupe of 3‐4 cm in length, ovoid, oblong and flat at the base, having thin and smooth skin, a thin pulp, a yellow‐orange colour and an acidic flavour. The fruit has a large white fibrous seed.^21^ All parts of the plant (ie, leaves, pulp and skin) are reported to be useful and are rich in beta‐cryptoxanthin, lutein, flavonoids and phenolic compounds.^22^ This unique composition showed antioxidant and anti‐inflammatory effects in clinical and experimental studies.^22^, ^23^, ^24^ However, the effects of SM supplementation in cardiovascular health are not well studied. Akinmoladun et al^25^ showed that supplementation with SM before cardiac injury induced by isoproterenol in rats protected against inflammation and oxidative stress. Interestingly, these effects were comparable with the effects of the angiotensin‐converting enzyme inhibitor ramipril.^25^

Despite these effects, there was no study investigating the effects of SM on cardiac remodelling induced by ETS. Thus, the objective of this study was to investigate the influence of SM supplementation on the cardiac remodelling process induced by ETS in rats.

## 2 MATERIALS AND METHODS

This research protocol was approved by the Animal Ethics Committee of Botucatu Medical School (1116‐2015), and it was performed in accordance with the National Institute of Health's Guide for the Care and Use of Laboratory Animals.

Male Wistar rats weighing 200‐250 g were divided into 4 groups: group C (control, n = 20) comprised animals not exposed to cigarette smoke and received standard chow; group ETS (exposed to tobacco smoke, n = 20) comprised animals exposed to cigarette smoke and received standard chow; group ETS100 (n = 20) animals were exposed to cigarette smoke and received standard chow supplemented with 100 mg/kg body weight/d of SM; and group ETS250 (n = 20) animals were exposed to cigarette smoke and received standard chow supplemented with 250 mg/kg body weight/d of SM.^25^ The rats were housed in individual cages, in a temperature‐controlled room (24°C) with a 12‐hour light/dark cycle. Water was supplied ad libitum. The dietary intake was recorded daily. The animals were observed for 2 months, during which morphological, biochemical and functional analyses were performed.

### 2.1 Chow preparation

The skin and pulp of SM were triturated, and the juice was maintained in a −80°C freezer. The total phenolic compounds, antioxidant activity and total carotenoids of the juice were analysed according to Kim et al, Brand‐Williams et al and Carvalho et al, respectively.^26^, ^27^, ^28^ Total phenolic compounds expressed as quantity of gallic acid were 96.74 mg/100 g; antioxidant activity was 12.84 g DPPH/kg; and total carotenoids, expressed as quantity of beta‐carotene, was 4.28 mg/100 mg. The total amount of water in the juice was determined (88.2%), and we added to the standard chow the equivalent of 100 mg (ETS100 group) and 250 mg (ETS250 group) of the dry extract/kg body weight/d. These doses of SM supplementations were determined according to Akinmoladun et al.^25^ Thus, for each kilogram of standard chow, 21.5 g and 54 g of the SM juice were added in the ETS100 and ETS250 groups, respectively.

The 100 and 250 mg of SM/kg body weight/d are equivalent to 329 and 610 g/d for a 60 kg human, respectively. For this calculation, we used the following formula described by Reagan‐Shaw et al^29^:

$$\begin{array}{cc} \text{Human}\;\text{equivalent}\;\text{doses}=\; & \text{Animal}\;\text{dose}\;(\text{mg}/\text{kg})\\ & *(\text{animal}\;\text{Km}/\text{human}\;\text{Km}) \end{array}$$

The Km factor, BW (kg) divided by BSA (m^2^), is used to convert the mg/kg dose used in a study to an mg/m^2^ dose (human Km of 60 kg: 37 and rat Km of 400 g: 7.7).

### 2.2 Exposure to tobacco smoke

The ETS rats were exposed to cigarette smoke in a chamber (dimensions 95 × 80 × 65 cm) connected to a smoking device constructed based on a model published by Wang et al and adapted by Paiva et al.^30^, ^31^ During the first week, the number of cigarettes was gradually increased from 5 to 10 cigarettes delivered in a 30‐min period, administered twice each afternoon. Subsequently, 10 cigarettes were used in each smoking trial, repeated 4 times per day, twice in the morning and twice in the afternoon. The cigarette composition per unit was 0.8 mg of nicotine, 10 mg of tar and 8 mg of carbon monoxide.

### 2.3 Echocardiographic analysis

After 2 months, all the rats were weighed and evaluated by a transthoracic echocardiographic examination (General Electric Medical Systems, Vivid S6, Tirat Carmel, Israel). The following structural variables were measured: left atrium (LA) diameter, diastolic and systolic left ventricle (LV) diameter (LVDD and LVSD, respectively) and LV diastolic posterior wall thickness (PWT). Systolic function was assessed based on endocardial fractional shortening and posterior wall shortening velocity (PWSV). The E/A ratio, the isovolumetric relaxation time and the isovolumetric relaxation time corrected by the heart rate (IRT/RR0.5) were used as indices of LV diastolic function.^32^

### 2.4 Isolated heart study: Langendorff preparation

After 2 months of cigarette exposure, 8 animals from each group were evaluated according to the previously described method. We registered the diastolic and systolic pressures, the maximum LV pressure decrease rate (−d*P*/d*t*) and the maximum LV pressure development rate (+d*P*/d*t*). Systolic function was evaluated by +d*P*/d*t* and diastolic function by −d*P*/d*t*. Developed pressure was also measured. The hearts that were subjected to the isolated heart study were not used for any other analysis, as retrograde perfusion can interfere with subsequent biochemical analysis.^33^

### 2.5 Morphometric analysis

The right and left ventricles (including the interventricular septum) were dissected and separated. Transverse sections of the LV were fixed in 10% buffered formalin and embedded in paraffin. Five‐micrometre‐thick sections were stained with haematoxylin and eosin. The myocyte CSA was determined as previously described.^13^

### 2.6 Cardiac LH and antioxidant enzyme analysis

Left ventricle samples (100 mg) were homogenized in 5 mL of 0.1 mol/L cold sodium phosphate buffer, pH 7.4, containing 1 mmol/L ethylene diamine tetra‐acetic acid. Tissue homogenates were prepared, and total protein concentration, glutathione peroxidase (GSH‐Px), superoxide dismutase (SOD) and catalase (CAT) were assessed as previously specified.^27^ All reagents were purchased from Sigma (St. Louis, MO, USA).^34^, ^35^, ^36^

### 2.7 Evaluation of serum cotinine

Serum cotinine levels were evaluated by enzyme‐linked immunosorbent assay (ELISA) according to the manufacturer's instructions (Sigma‐Aldrich; product #SE120083). The sensitivity of the ELISA kit was 1 ng/mL.

### 2.8 Energy metabolism enzymes

Cardiac energy metabolism was assessed by β‐hydroxyacyl coenzyme‐A dehydrogenase (OHADH, EC 1.1.1.35.), lactate dehydrogenase (LDH, EC 1.1.1.27), citrate synthase (CS; EC 4.1.3.7.), phosphofructokinase (PFK), pyruvate dehydrogenase (PD), complex I (NADH: ubiquinone oxidoreductase), complex II (succinate dehydrogenase) and ATP synthase (EC 3.6.3.14) activities, as previously described.^37^ Spectrophotometric determinations were performed with a Pharmacia Biotech spectrophotometer UV/visible Ultrospec 5000 with Swift II Application software (Cambridge, England, UK) at 560 nm. All reagents were purchased from Sigma.

### 2.9 Western blot analysis for Nrf‐2, SIRT‐1, NF‐κB, IFN‐γ, IL‐10, type I and III collagen

Briefly, samples of LV were homogenized in RIPA buffer and diluted in Laemmli buffer to detect type I collagen (rabbit polyclonal IgG, sc8784R, Santa Cruz Biotechnology, Inc., Dallas, TX, USA), type III collagen (mouse monoclonal IgG1, ab6310R; Abcam, Inc., MA, USA), SIRT‐1 (rabbit polyclonal IgG, sc15404; Santa Cruz Biotechnology, Inc., Europe), IL‐10 (rat monoclonal IgG, ab33471; Abcam Inc.), INF‐γ (mouse monoclonal IgG1, ab133566; Abcam Inc.), total NF‐κB (mouse monoclonal IgG, sc8008) and phosphorylated NF‐κB (rabbit monoclonal IgG, sc3302; Santa Cruz Biotechnology, Inc., Europe). Nuclear protein extraction from the LV was performed with the NE‐PER Nuclear Extraction Reagents kit (Pierce Biotechnology, Rockford, IL, USA). Nuclear extracts were used to detect Nrf‐2 (C‐20, rabbit polyclonal IgG, sc722; Santa Cruz Biotechnology, Inc., Europe). Secondary antibodies were used according to the manufacturer's recommendations, and GAPDH (GAPDH [6C5], mouse monoclonal IgG1, sc32233; Santa Cruz Biotechnology, Inc., Europe) was used for normalization.^37^

### 2.10 Statistical analysis

Comparisons among groups were performed by 1‐way analysis of variance (ANOVA) and the Tukey test for parameters with normal distribution. Otherwise, comparisons among groups were made using the Kruskal‐Wallis test and the Dunn post‐test. Normality as assessed with the Shapiro‐Wilk test. Data were expressed as the mean ± SD or medians (including the lower quartile and upper quartile). For mortality comparison, we used the chi‐square test. Data analysis was performed with SigmaStat for Windows v2.03 (SPSS Inc, Chicago, IL, USA). The significance level was 5%.

## 3 RESULTS

Only 1 animal (in the ETS250 group) died during the follow‐up period. There was no difference among body weights in the beginning of the study; however, the groups exposed to cigarette smoke had lower body weight at the end of the experiment compared with the control group (Table 1). In addition, chow ingestion was lower in animals exposed to cigarette smoke (C group [n = 20]: 20.5 ± 0.2 g; ETS group [n = 20]: 19.9 ± 0.7 g; ETS100 group [n = 20]: 19.7 ± 0.6 g; and ETS250 group [n = 20]: 19.8 ± 0.5 g; *P* < .001). Serum cotinine levels, as expected, were increased in the ETS groups compared with the control group. There were no differences among the ETS groups (C group [n = 20]: 0.0 ± 0.0 ng/mL; ETS group [n = 20]: 56.6 ± 17.6 ng/mL; ETS100 group [n = 20]: 50.6 ± 15.7 ng/mL; and ETS250 group [n = 20]: 57.0 ± 16.6 g; *P* = .004). This result confirms the efficacy of the procedure for exposing animals to cigarette smoke.

**Table 1:** Morphological and functional data evaluated by echocardiography

Variables	C group (n = 20)	ETS group (n = 20)	ETS100 group (n = 20)	ETS250 group (n = 20)	*P* value
BW (g)	411 (405‐423)	369 (339‐394)^a^	361 (349‐391)^a^	366 (354‐404)^a^	<.001
HR (bpm)	313.1 ± 54.1	302.1 ± 50.0	298.0 ± 33.7	302.5 ± 51.2	.781
LA area (cm^2^)	22.0 ± 3.8	25.6 ± 5.2^a^	24.1 ± 2.8	22.2 ± 3.5^b^	.017
LA/BW (mm/kg)	11.4 (11.1‐12.4)	14.6 (13.1‐16.0)^a^	14.6 (13.6‐15.1)^a^	12.8 (12.2‐15.0)^a^	<.001
LVDD/BW (mm/kg)	16.8 ± 1.1	20.3 ± 2.6^a^	19.0 ± 1.5^a^	18.5 ± 1.6^a^^,^ b	<.001
LVSD/BW (mm/kg)	6.8 (6.0‐7.2)	8.4 (7.3‐11.1)^a^	8.8 (7.5‐9.8)^a^	8.2 (7.5‐8.7)^a^	<.001
RWT	0.47 ± 0.05	0.43 ± 0.07	0.46 ± 0.06	0.48 ± 0.05	.134
IMVE (g/kg)	1.8 (1.5‐2.0)	2.1 (1.9‐2.4)^a^	2.0 (1.8‐2.2)	2.0 (1.9‐2.3)	.012
EF (%)	0.93 (0.90‐0.95)	0.91 (0.86‐0.94)	0.91 (0.89‐0.93)	0.92 (0.89‐0.93)	.156
FS (%)	58.6 (54.1‐62.5)	55.4 (48.4‐61.4)	54.8 (52.1‐59.3)	56.0 (52.0‐58.4)	.157
IRT/RR^0.5^ (ms)	0.09 ± 0.02	0.09 ± 0.03	0.08 ± 0.02	0.09 ± 0.02	.563
E/A	1.6 (1.4‐1.7)	1.7 (1.5‐2.0)	1.8 (1.5‐2.0)	1.7 (1.5‐1.8)	.231
EDT (ms)	49.2 ± 5.3	48.4 ± 8.4	48.7 ± 5.1	50.2 ± 4.5	.790

BW, body weight; E/A, peak velocity of early ventricular filling/peak velocity of transmitral flow during atrial contraction; EDT, E‐wave deceleration time; EF, ejection fraction; ETS, exposed to tobacco smoke; FS, endocardial fractional shortening; HR, heart rate; IRT/RR^0.5^, isovolumetric relaxation time adjusted by heart rate; LA, left atrium; LVDD, LV end‐diastolic dimension; LVSD, LV end‐systolic dimension; RWT, relative wall thickness.

Data are expressed as mean ± SD or as the median (lower quartile‐upper quartile). One‐way ANOVA/Tukey.

a ≠ with C group.

b ≠ with ETS group.

The echocardiographic data are shown in Table 1. The ETS animals (ETS, ETS100 and ETS250) had higher values of LVDD/BW, LVSD/BW and AE/BW compared with the C group. In addition, ETS250 group had a lower value of LVDD/BW than the ETS group. Regarding the LA area and LVMI, only the ETS group had higher values than the C group, and the ETS250 group had lower LA area than the ETS group. There were no differences regarding echocardiographic functional variables among groups (Table 1). The results of the isolated heart study were also similar among groups (Table 2).

**Table 2:** Isolated heart data

Variables	C group (n = 20)	ETS group (n = 20)	ETS100 group (n = 20)	ETS250 group (n = 20)	*P* value
Systolic pressure (mm Hg)	130.5 ± 12.5	130.6 ± 14.5	126.9 ± 21.5	133.4 ± 28.5	.935
+d*P*/d*t* max (mm Hg/s)	2375 ± 378	2469 ± 382	2359 ± 599	2661 ± 874	.743
−d*P*/d*t* max (mm Hg/s)	1797 ± 238	1914 ± 315	1734 ± 414	2000 ± 629	.612
DP (mm Hg)	99.2 ± 11.5	104.0 ± 16.4	96.9 ± 20.7	104.4 ± 27.7	.846

+d*P*/d*t* max, maximum left ventricular pressure development rate; −d*P*/d*t* max, maximum left ventricular pressure decrease rate; DP, developed pressure; ETS, exposed to tobacco smoke.

Data are expressed as mean ± SD. One‐way ANOVA/Tukey.

The morphological data are summarized in Table 3. LV/BW was higher in the ETS group compared with the C group. The animals in the ETS100 and ETS250 groups had values of LV/BW similar to C group. In addition, the CSA was lower in the C group compared with the ETS groups; however, ETS250 had lower values of CSA compared with the ETS group.

**Table 3:** Morphometrical analysis

Variables	C group (n = 20)	ETS group (n = 20)	ETS100 group (n = 20)	ETS250 group (n = 20)	*P* value
LV (g)	0.87 ± 0.11	0.90 ± 0.14	0.84 ± 0.09	0.86 ± 0.11	.500
RV (g)	0.23 (0.20‐0.27)	0.24 (0.19‐0.27)	0.20 (0.19‐0.23)	0.22 (0.19‐0.27)	.370
LV/BW	2.1 ± 0.3	2.5 ± 0.5^a^	2.3 ± 0.2	2.3 ± 0.3	.005
RV/BW	0.56 (0.49‐0.64)	0.63 (0.52‐0.86)	0.56 (0.52‐0.64)	0.61 (0.53‐0.65)	.332
CSA^c^ (μm^2^)	266.6 ± 23.2	347.5 ± 15.1^a^	302.5 ± 5.2^a^	294.7 ± 8.6^a^^,^ b	<.001

BW, body weight; CSA, myocyte cross‐sectional area; ETS, exposed to tobacco smoke; LV, left ventricle; RV, right ventricle.

Data are expressed as mean ± standard deviation (SD) or as the median (lower quartile‐upper quartile). One‐way ANOVA/Tukey.

a ≠ with C group.

b ≠ with ETS group.

c CSA analysis performed in 6 animals per group.

Oxidative stress data and antioxidant enzyme activity are shown in Table 4. The ETS group had higher cardiac levels of LH compared with the C group; and, ETS100 and ETS250 had lower concentrations of LH compared with the ETS group. The cardiac activity of CAT was lower in animals exposed to cigarette smoke. In addition, the activity of SOD and GSH‐Px was lower in the ETS group compared with the C group, and the supplementation of SM increased these activities in the ETS100 and ETS250 groups compared with the ETS group (Table 4).

**Table 4:** Left ventricle oxidative stress

Variables	C group (n = 8)	ETS group (n = 8)	ETS100 group (n = 8)	ETS250 group (n = 8)	*P* value
LH (nmol/g)	196.4 ± 51.5	331.9 ± 52.9^a^	207.2 ± 53.0^b^	165.1 ± 40.3^b^	<.001
CAT (μmol/g)	75.8 ± 7.8	54.0 ± 7.3^a^	59.6 ± 5.8^a^	61.1 ± 6.5^a^	<.001
SOD (nmol/g)	7.9 ± 0.4	5.7 ± 0.4^a^	7.5 ± 0.9^b^	7.8 ± 0.9^b^	<.001
GSH‐Px (nmol/g)	39.2 ± 5.6	18.8 ± 3.4^a^	30.0 ± 5.6^a^^,^ b	31.1 ± 4.3^a^^,^ b	<.001

CAT, catalase; ETS, exposed to tobacco smoke; GSH‐Px, glutathione peroxidase; LH, lipid hydroperoxide; SOD, superoxide dismutase.

Data are expressed as mean ± SD. One‐way ANOVA/Tukey.

a ≠ with C group.

b ≠ with ETS group.

Regarding energy metabolism analysis, the data are presented in Table 5. The ETS group had lower values of OHADH compared with the C group and with the ETS100 group. The activity of PFK and LDH was higher in the ETS and ETS100 groups compared with the C group; however, the ETS250 group had lower activity of these enzymes compared with the ETS and ETS100 groups. There were no differences in PD activity among groups. The animals exposed to cigarette smoke had lower values of CS, complex I and ATP synthase compared with the C group. The ETS250 group had higher values of CS compared with the ETS group and of complex I compared with the ETS and ETS100 groups. Regarding complex II activity, it was higher in the C and ETS250 groups compared with the ETS and ETS100 groups.

**Table 5:** Energy metabolism data

Variables	C group (n = 8)	ETS group (n = 8)	ETS100 group (n = 8)	ETS250 group (n = 8)	*P* value
OHADH (nmol/mg)	33.2 ± 7.8	20.4 ± 4.0^a^	32.5 ± 7.3^b^	28.3 ± 7.0	.002
PFK (nmol/g)	135.9 ± 22.8	188.3 ± 22.2^a^	188.3 ± 21.7^a^^,^ c	148.5 ± 19.0^b^	<.001
LDH (nmol/mg)	85.1 ± 8.8	140.1 ± 10.2^a^	134.8 ± 12.9^a^^,^ c	96.8 ± 12.2^b^	<.001
PD (nmol/g)	211.4 ± 32.2	169.3 ± 23.2	178.2 ± 40.6	198.3 ± 33.9	.067
CS (nmol/mg)	85.6 ± 8.1	35.7 ± 8.1^a^	42.8 ± 3.4^a^	52.5 ± 8.6^a^^,^ b	<.001
Complex I (nmol/mg)	10.3 ± 1.4	2.8 ± 0.4^a^	3.3 ± 0.4^a^^,^ c	8.3 ± 1.2^a^^,^ b	<.001
Complex II (nmol/mg)	3.7 ± 0.3	1.9 ± 0.4^a^	2.1 ± 0.4^a^^,^ c	3.4 ± 0.2^b^	<.001
ATP synthase (noml/mg)	32.3 ± 2.4	19.4 ± 2.5^a^	19.5 ± 3.0^a^	22.0 ± 3.1^a^	<.001

CS, citrate synthase; ETS, exposed to tobacco smoke; LDH, lactate dehydrogenase; OHADH, β‐hydroxyacyl coenzyme‐A dehydrogenase; PD, pyruvate dehydrogenase; PFK, phosphofructokinase.

Data are expressed as mean ± SD. One‐way ANOVA/Tukey.

a ≠ with C group.

b ≠ with ETS group.

c ≠ with ETS250 group.

**Figure 1:**
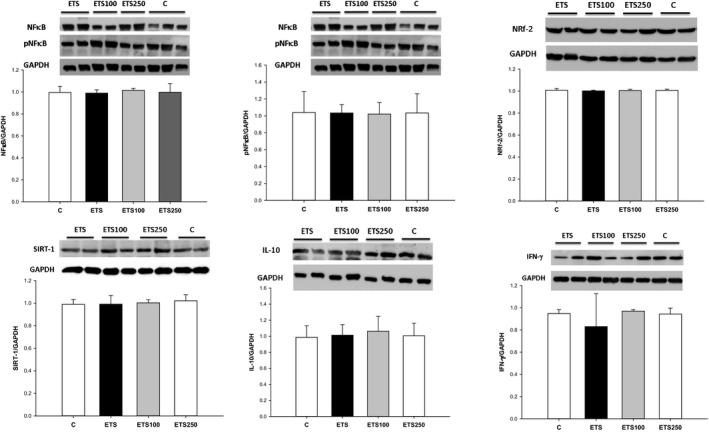
Western blot analysis of cardiac Nrf‐2, SIRT‐1, NF‐κB, IFN‐γ and IL‐10 among groups

**Figure 2:**
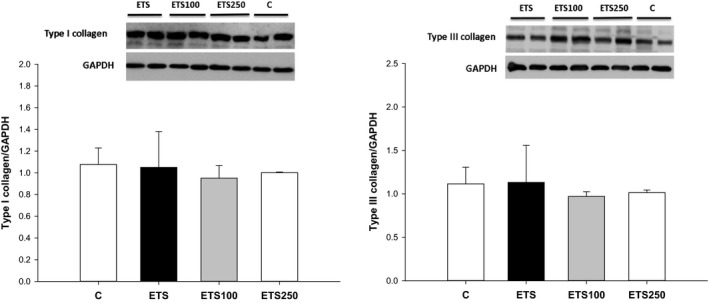
Western blot analysis of cardiac types I and III collagen among groups

In the Western blot analysis, there were no differences in the expression of Nrf‐2, SIRT‐1, NF‐κB, IFN‐γ, IL‐10 and types I and III collagen among groups (Figures 1 and 2).

## 4 DISCUSSION

The objective of our study was to analyse the influence of SM supplementation on the cardiac remodelling process induced by ETS in rats. Our data showed that ETS induced the cardiac remodelling process. In addition, SM supplementation attenuated this process, along with oxidative stress reduction and energy metabolism modulation.

Tobacco smoke can lead to cardiovascular diseases due to its effects on the vascular system and direct effects on cardiac remodelling.^5^, ^6^, ^7^, ^8^, ^38^, ^39^ In our study, as expected, ETS increased LV chamber diameter and induced myocyte hypertrophy. However, we did not observe functional alterations in echocardiogram or in isolated heart analysis, as we previously reported.^10^, ^11^, ^14^, ^15^, ^17^, ^18^ Because the time of tobacco exposure was the same (2 months), we believe that alterations in cigarette content, in the last years, such as reduction in nicotine and tar content, could explain our results. Despite that, serum cotinine levels were increased in animals exposed to tobacco smoke. In addition, morphological and biochemical alterations were observed with our protocol. These alterations are characteristics of the cardiac remodelling process.

Due to the high socio‐economic impact and the high mortality rates, it is relevant to identify factors that modulate the cardiac remodelling process. In this scenario, we can highlight the supplementation of substances with anti‐inflammatory and antioxidant properties, such as SM. In our study, the chemical analysis we performed showed higher amounts of carotenoids, antioxidants and phenolic content in SM extract. Other studies also determined SM content. Tiburski et al showed that beta‐cryptoxanthin is the most important carotenoid found in SM, with approximately 65 μg/250 g of SM.^23^ In addition, Vasco et al^40^ found values of total phenolic content of 249 mg/100 g of SM, and suggested that gallic acid, hydroxycinnamic acid and caffeic acid are the major phenolic compounds of SM. In our study, the supplementation of SM, mainly at the higher dose, attenuated the morphological alterations induced by ETS. We believe the synergism between these compounds could modulate these effects.

Studies evaluating SM supplementation and cardiovascular health are scarce. One study evaluated the effects of SM leaf extract supplementation using the in vitro Langendorff technique and the in vivo isoproterenol‐induced myocardial infarction model and compared them with ramipril. The authors showed that SM improved cardiac function, preserved the disruption of cardiac myofibrils and the myocyte membrane integrity and reduced oxidative stress. In addition, these results were comparable with the effects of ramipril. Despite these beneficial effects, SM supplementation was not yet evaluated in the ETS model.

Tobacco smoke also induces oxidative stress. Oxidative stress is defined as an imbalance between the production of reactive oxygen species (ROS) and reactive nitrogen species and the body's antioxidant defences. Regarding the enzymatic antioxidant defence system, CAT, SOD and GHS‐Px deserve attention. In fact, previous studies have suggested that ETS increases NADPH oxidase activity and ROS formation.^5^, ^6^ At the micromolar level, ROS are generally believed to be harmful because they cause oxidative damage to protein, DNA and lipids.^14^ At nanomolar concentrations, they function as a second messenger in signalling pathways and modulate transcription factors such as Nrf‐2, SIRT‐1 and NFκ‐B.

Nrf‐2 is an important transcription factor that coordinates the expression of antioxidant response element‐containing genes, including SOD, GSH‐Px, heme oxygenase‐1 and many enzymes involved in glutathione production.^41^ It is interesting to observe that some studies suggest that the Nrf‐2 pathway could also be stimulated by SIRT‐1.^41^, ^42^ SIRT‐1 is a nicotinamide adenosine dinucleotide (NAD^+^)‐dependent protein deacetylase that possesses remarkable antioxidant capacity by deacetylating numerous substrates. Thus, these pathways are usually up‐regulated to protect the organisms from ROS damage.^42^ However, in our study, the expression of these proteins was not increased with ETS.

NFκ‐B is a key regulator in the inflammatory pathway, and its activation triggers the expression of downstream target genes, including inflammatory cytokines, chemokines and adhesion molecules. In our study, we also did not find alterations in pro‐inflammatory cytokines or in the NFκ‐B expression, suggesting that inflammation is not as important as oxidative stress in this model of tobacco smoke exposure.

Alterations in energy metabolism were also important mechanisms in the cardiac remodelling process induced by tobacco exposure. Under physiological conditions, free fatty acids are the main energy substrate of the heart, accounting for 60%‐90% of the energy supply.^43^, ^44^ Fatty acids and glucose metabolites enter the citric acid cycle by β‐oxidation and glycolysis, respectively, to generate FADH2 and NADH, which participate in the electron transport chain. In the pathological condition, the heart switches the fuel source from fatty acids to glucose, lactate and pyruvate. This shift can be observed through the increased activity of PFK, LDH and PD, enzymes of the glycolytic pathway, and the decreased activity of OHADH involved in the β‐oxidative process.^43^, ^44^ We found these alterations in ETS rats. In addition, we also showed a reduction in energy production due to the decreased activity of complex I, II and ATP synthase. The SM supplementation decreased glycolysis and increased the β‐oxidation and the oxidative phosphorylation. It is also important to observe that these effects were greater with the higher dose of SM supplementation.

In this study, we showed that SM supplementation, mainly at higher doses, attenuated the cardiac remodelling process induced by ETS. This effect was associated with reduction in oxidative damage and modulation of energy metabolism. We speculate that the synergic interaction between carotenoids, flavonoids and phenolic compounds could be responsible for these beneficial effects.

In conclusion, our results suggest that ETS induced the cardiac remodelling process. In addition, SM supplementation attenuated this process, along with oxidative stress reduction and energy metabolism modulation.

## CONFLICT OF INTERESTS

The authors confirm that there are no conflict of interests.

## AUTHORS’ CONTRIBUTIONS

MAML, BLBP involved in acquisition, analysis and interpretation of data and drafting the article. MGB, AGA, AMF, RACS, EMK, PHCA, TFB, SGZ, PSA, BFP, AAHF involved in acquisition, analysis and interpretation of data. LAMZ, SARP, MFM performed study design, data interpretation and revising the manuscript. All authors revised the article critically for important intellectual content and approved the final version of the manuscript.
